# Mitochondrial genome evolution in fire ants (Hymenoptera: Formicidae)

**DOI:** 10.1186/1471-2148-10-300

**Published:** 2010-10-07

**Authors:** Dietrich Gotzek, Jessica Clarke, DeWayne Shoemaker

**Affiliations:** 1 Department of Ecology and Evolution, University of Lausanne, 1015 Lausanne, Switzerland; 2 Department of Entomology, University of Wisconsin, Madison, Wisconsin, USA; 3 USDA-ARS Center for Medical, Agricultural, and Veterinary Entomology, 1600/1700 SW 23rd Drive, Gainesville, Florida, USA

## Abstract

**Background:**

Complete mitochondrial genome sequences have become important tools for the study of genome architecture, phylogeny, and molecular evolution. Despite the rapid increase in available mitogenomes, the taxonomic sampling often poorly reflects phylogenetic diversity and is often also biased to represent deeper (family-level) evolutionary relationships.

**Results:**

We present the first fully sequenced ant (Hymenoptera: Formicidae) mitochondrial genomes. We sampled four mitogenomes from three species of fire ants, genus *Solenopsis*, which represent various evolutionary depths. Overall, ant mitogenomes appear to be typical of hymenopteran mitogenomes, displaying a general A+T-bias. The *Solenopsis *mitogenomes are slightly more compact than other hymentoperan mitogenomes (~15.5 kb), retaining all protein coding genes, ribosomal, and transfer RNAs. We also present evidence of recombination between the mitogenomes of the two conspecific *Solenopsis *mitogenomes. Finally, we discuss potential ways to improve the estimation of phylogenies using complete mitochondrial genome sequences.

**Conclusions:**

The ant mitogenome presents an important addition to the continued efforts in studying hymenopteran mitogenome architecture, evolution, and phylogenetics. We provide further evidence that the sampling across many taxonomic levels (including conspecifics and congeners) is useful and important to gain detailed insights into mitogenome evolution. We also discuss ways that may help improve the use of mitogenomes in phylogenetic analyses by accounting for non-stationary and non-homogeneous evolution among branches.

## Background

There has been a rapid proliferation of whole mitochondrial genomes (mitogenomes) sequenced in recent years, no doubt driven in part by the increasing speed and decreasing cost of sequencing technologies. Whole mitogenomes are increasingly used in phylogenetic studies [1-6] and in analyses of genome rearrangements [7-11], which can also be used for phylogenetic inference [8,12-16].

**Figure 1:**
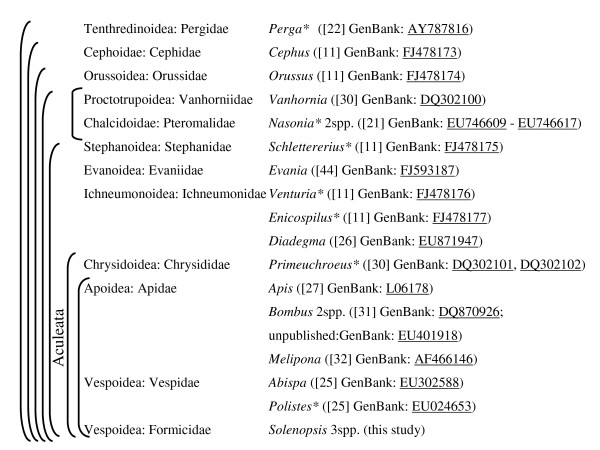
**Available hymenopteran mitogenomes ordered phylogenetically (based on **[11,54]**)**. Parentheses indicate monophyly, superfamilies and families are assumed to be monophyletic. Asterices indicates partially sequenced genomes. Citations and GenBank accession numbers are also given.

However, the utility of these datasets for these purposes greatly depends on taxon sampling. Currently, 237 insect mitogenomes have been fully sequenced (GenBank Sept. 21, 2009), yet the taxa utilized for these sequencing studies often do not reflect the distribution of species diversity. For example, Hymenoptera is one of the most species-rich insect orders (~130,000 described species in 22 superfamilies [17]), yet only 11 mitogenomes have been fully sequenced (compared to 28 for beetles [300,000 species] and 69 for flies [110,000 species]). Despite a further seven hymenopteran mitogenomes being partially sequenced (Figure 1), taxon sampling still poorly reflects phylogenetic diversity of this important order across many taxonomic levels. Naturally, sampling is highly dependent on the questions a given researcher wishes to address with the data, yet biased sampling greatly limits the utility of the generated mitogenomic data in a comparative framework. For example, of the ten families of bees (Apoidea) and of the nine families of vespids (Vespoidea) only a single family (Apidae and Vespidae, respectively) of each superfamily has a sequenced mitogenome. But not only are many taxonomically and ecologically important families unsampled, there is also a dearth of mitogenomes for closely related species. We follow Gissi et al. [18] in arguing that to better understand mitochondrial genome evolution we require an improved taxon sampling scheme that not only captures phylogenetic diversity more broadly but also takes into account various evolutionary depths, including variation within or among closely related species.

Hence, we here present the first complete mitogenomes of ants (Hymentopera: Formicidae; four mitogenomes from three fire ant species) and discuss their evolution in light of the other hymenopteran mitochondrial genomes. The four mitogenomes presented here represent important contributions to the ever expanding dataset of complete hymenopteran mitochondrial genomes in that they represent a previously unsampled, highly diverse, and ecologically dominant vespoid family (Formicidae). Our mitogenome data for three fire ant species belonging to two species groups [19,20] also provides insights into mitogenome evolution at lower taxonomic levels. Such data generally are rare [18], with the notable exceptions in insects of a study employing comparable sampling for *Nasonia *[21] and much more extensive sampling in *Drosophila *(38 mitogenomes; GenBank Sept. 21, 2009).

## Results and Discussion

For the present study we sequenced four complete mitogenomes from three fire ant species. These included two mitogenomes from *Solenopsis invicta *Buren, 1972 (referred to hereafter as "PMS" and "VMS" mitogenomes), one mitogenome from the closely related species *S. richteri *Buren, 1972 ("*richteri*" mitogenome) and one mitogenome from a more distantly related fire ant species *S. geminata *(Fabricius, 1804) ("*geminata*" mitogenome). These first complete mitogenome data for ants expand taxon selection of one of the largest and most diverse hymenopteran superfamilies (Vespoidea).

At first look, the mitogenomes of *Solenopsis *appear to be unremarkable compared with other hymenopteran mitogenomes, containing the same complement of protein-coding loci, tRNAs, rRNAs, and a pronounced A+T-bias (Table 1). Overall the ant mitogenomes are less divergent than those of *Nasonia *(3 - 13% uncorrected nucleotide distance, π, compared with ~15% among jewel wasps), however this comparison should be viewed with caution since it does not consider the potential differences in age among species within the *Nasonia *and *Solenopsis *species groups, which is not known. Also, we found no clear evidence of positive, directional selection acting on the ant mitogenomes (dN/dS ≈ 0.001 - 0.034 for the 13 protein coding genes using the site model [22,23]).

**Table 1:** Summary of *Solenopsis *mitogenomes.

	length (bp)	PCG+rRNA	tRNAs	overall AT bias	protein AT bias	ENC	CBI	scaled **χ**^**2**^
VMS	15,548	13+2	22	77.5	74.5	42.971	0.563	0.103
PMS	15,549	13+2	22	77.2	74.2	43.270	0.543	0.106
*richteri*	15,560	13+2	22	76.9	74.0	43.477	0.534	0.099
*geminata*	15,552	13+2	22	76.5	73.5	44.695	0.524	0.126

PCG: protein coding genes; ENC: effective number of codons (out of a maximum of 61) [92]; CBI: Codon Bias Index [91]. The scaled χ^2 ^[93] used to test for codon bias was corrected for G+C bias using Yates' correction.

### Gene content and order

**Figure 2:**
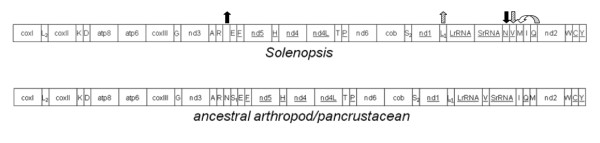
**Schematic of gene order in the *Solenopsis *mitogenomes compared to the ancestral arthropod/hymenopteran mitogenome**. All protein-coding genes and rRNAs are oriented in the same direction as all other hymenopterans and the hypothetical ancestral arthropod mitogenome. Underlined loci indicate location on the N strand. Arrows are coded by hatching and indicate changes in tRNA order relative to the ancestral arthropod mitogenome.

Consistent with other published hymenopteran mitogenomes, the four *Solenopsis *mitogenomes contain all 13 protein-coding genes and both rRNAs in the same order and direction of the hypothesized ancestral pancrustacean mitogenome (Figure 2). As in the three *Nasonia *species [21], the four *Solenopsis *mitogenomes share identical architectures at the tRNA loci as well. However, the three methods we used to identify tRNAs greatly differed in sensitivity and accuracy (Table 2). Both DOGMA (used at COVE cut-off score = 20) and ARWEN over-predicted tRNAs (usually well over 30 for DOGMA and up to 26 by ARWEN). As a result, they usually identified more of the 22 tRNAs than tRNAscan-SE. tRNAscan-SE was more conservative, although some predictions were not well supported (COVE scores < 20). It also misidentified tRNAs three times: two tRNA-F in *geminata *(COVE = 19.47) and at the same position in VMS (COVE = 18.65) and tRNA-P (COVE = 23.85) also in VMS. Since all of these tRNAs overlap with *SrRNA *(tRNA-F) or *nad5 *(tRNA-P) and have relatively low COVE scores, we considered them false positives. Only twice were none of the methods able to detect tRNAs: tRNA-S_1 _in *geminata *and tRNA-N in VMS. However, we were able to manually fold these tRNAs.

**Table 2:** Comparison of various methods to identify tRNAs in the *Solenopsis *mitogenomes.

		*geminata*			*Richteri*			PMS			VMS		
		
		DOGMA	tRNAscan	ARWEN	DOGMA	tRNAscan	ARWEN	DOGMA	tRNAscan	ARWEN	DOGMA	tRNAscan	ARWEN
tRNA Type	Anti Codon	Cove score	Cove Score		Cove score	Cove Score		Cove score	Cove Score		Cove score	Cove Score	
L_2_	TAA	47.63	22.63	yes	49.15	31.79	yes	49.15	31.79	yes	49.15	31.06	yes
K	TTT	44.74	18.29	yes	39.21	21.78	yes	39.21	21.78	yes	37.64	21.78	yes

D	GTC	47.83	24.42	yes	41.13	18.89	yes	42.77	18.09	yes	42.79	18.9	yes
G	TCC	58.63	36.52	yes	49.5	32.45	yes	51.34	29.97	yes	48.23	29	yes

A	TGC	46.06	19.45	yes	48.9	24.57	yes	48.9	24.57	yes	54.71	25.98	yes
R	TCG	45.47	19.34	yes	39.41	---	yes	39.41	---	yes	38.96	---	yes

S_1_	TCT	---	---	---	26.43	---	yes	26.43	---	yes	27.89	---	---
E	TTC	53.66	25.17	yes	52.91	24.39	yes	52.91	24.39	yes	52.91	24.39	yes

F	GAA	46.74	30.61	yes	38.05	24.63	yes	38.49	28.14	yes	38.26	---	yes
H	GTG	54.21	20.57	yes	43.51	18.42	yes	43.51	18.42	yes	43.51	18.42	yes

T	TGT	58.18	27.74	yes	53.59	29.42	yes	53.59	29.42	yes	58.58	24.55	yes
P	TGG	51.68	26.94	yes	56.84	22.42	yes	56.84	22.42	yes	59.46	25.1	yes

S_2_	TGA	48.14	20.98	yes	48.14	20.98	yes	48.14	20.98	yes	48.14	20.98	yes
L_1_	TAG	25.07	18.66	yes	19.23	17.8	---	20.18	18.97	yes	---	16.85	yes

N	ATT	25.55	15.34	---	32.4	---	---	32.4	---	yes	---	---	---
V	TAC	48.34	18.03	yes	59.55	33.94	yes	59.55	33.94	yes	55.58	31.31	yes

M	CAT	42.75	18.84	yes	44.28	15.68	yes	43.92	---	yes	42.53	20.11	yes
I	GAT	36.35	---	yes	36.59	15.13	yes	36.18	19.8	yes	37.02	21.35	yes

Q	TTG	53.61	42.39	yes	51.22	41.64	yes	51.22	41.64	yes	53.25	42.31	yes
W	TCA	52.16	27.57	yes	47.34	30.06	yes	48.69	27.06	yes	44.83	26.98	yes

C	GCA	56.88	28.08	yes	61.16	27.44	yes	57.83	26.43	yes	57.44	28.93	yes
Y	GTA	44.94	20.78	yes	46.2	26.86	yes	43.75	24.44	yes	47	24.81	yes

DOGMA and ARWEN generally overpredicted tRNAs (data not shown) and tRNAscan underpredicted tRNA loci. COVE scores >20 are generally considered to indicate reliable identification of tRNAs. Some tRNAs were readily identified by all methods, whereas some proved harder to identify and had to be manually folded in some mitogenomes (e.g., tRNA-S_1_). Loci underlined are located on the N strand.

The location of tRNAs differs from the hypothetical ancestral hymenopteran mitogenome [24], which is also a typical feature of hymenopteran mitogenome architecture [11,25]. However, with only three apparent translocations, the *Solenopsis *mitogenome architecture appears to be less derived than that of the other hymenopteran mitogenomes [11,25-32]. Most mitogenome rearrangements in Hymenoptera appear to be selectively neutral and involve tRNA translocations around the *coxII *- *atp8 *junction [29,30] and the *nd3 *- *nd5 *junction [11,25]. The *Solenopsis *mitogenomes show no variation in the *coxII *- *atp8 *junction and a translocation of tRNA-N from the *nd3 *- *nd5 *junction to the *SrRNA *- *nd2 *junction. The tRNA-V translocation also moved to the *SrRNA *- *nd2 *junction. Various types of gene order rearrangements are generally recognized, differing by their location (local vs. distant) and whether they retain their original orientation (inverted vs. not inverted) and generally can be classified into one of several categories: local inversions, local translocations (gene shuffling), translocations, and remote inversions (an inverted translocation) [25].

**Figure 3:**
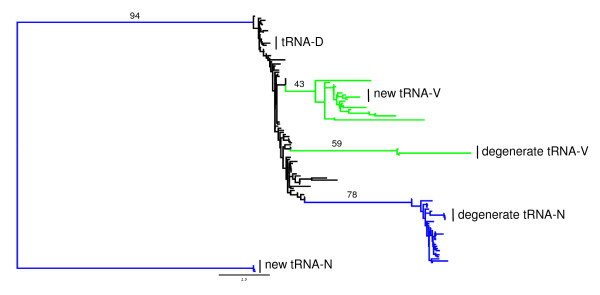
**Maximum likelihood phylogeny of hymenopteran tRNAs**. Only the *Solenopsis *tRNAs are labeled. Black branches identify tRNA-D loci. Blue identify all tRNA-N loci, including "new" and "degenerated" loci in *Solenopsis*. Green branches are tRNA-V loci, again with the "new" and "degenerated" loci labeled. The values above the long major branches are bootstrap support (100 replicates).

Visual inspection of the source locations for these two translocations led us to test the manner of the rearrangement, since short sequences of the length of typical tRNAs (approximately 60-80 bp) remained at the source locations (hereafter termed "degenerate" tRNAs). A duplication/loss model of translocation seems plausible for the tRNA-V translocation, whereas this model seems less likely for the tRNA-N rearrangement, since this rearrangement also involves an inversion (remote inversion). We consider an intra-mitochondrial recombination event [28] an unlikely mechanism in this particular case as well, since the translocation spanned half of the mitogenome and none of the intermediate genes are inverted or rearranged. Since DOGMA and ARWEN had placed an additional tRNA-N in the same position but opposite orientation as the tRNA-D in the VMS mitogenome (data not shown), we included all hymenopteran tRNA-N, -V, and -D loci in a phylogenetic analysis with the relevant *Solenopsis *tRNAs. The resulting tree placed the tRNA-D, tRNA-V, and degenerate tRNA-N firmly among their respective tRNA species, whereas the degenerate tRNA-V and new tRNA-N are clearly not closely related to any other tRNAs (Figure 3). This suggests that the identification of the overlapping tRNA-N over tRNA-D in VMS was erroneous. Also, the "new" translocated tRNA-V is clearly homologous to the other hymenopteran tRNA-V genes, suggesting a real translocation event. However, the identity of the degenerate locus is less clear. Since it forms a distinct and highly supported clade, we are inclined to interpret this as a duplication/loss translocation and the "degenerate" tRNA-V as a vestigial spacer, which we initially erroneously hypothesized to be a degenerate tRNA due to its position and length. Finally, the source of the tRNA-N is not clear and at present since we are unable to determine the source tRNA in our dataset (data not shown), but it is becoming increasingly clear that our understanding of tRNA evolution is rapidly changing [33-43]. We will require better sampling of other mitogenomes to shed light on the evolution of this tRNA.

Visual inspection of tRNAs across species reveals that there is considerable amount of variation due to point mutations and indels in almost all tRNAs. This variation results in differences in ability of the three tRNA identification methods to correctly identify tRNAs across species (Table 2), suggesting that congeneric comparative studies of tRNAs are fruitful endeavors to studying tRNA evolution and mitgenome architecture [18].

### Nucleotide and codon bias

**Figure 4:**
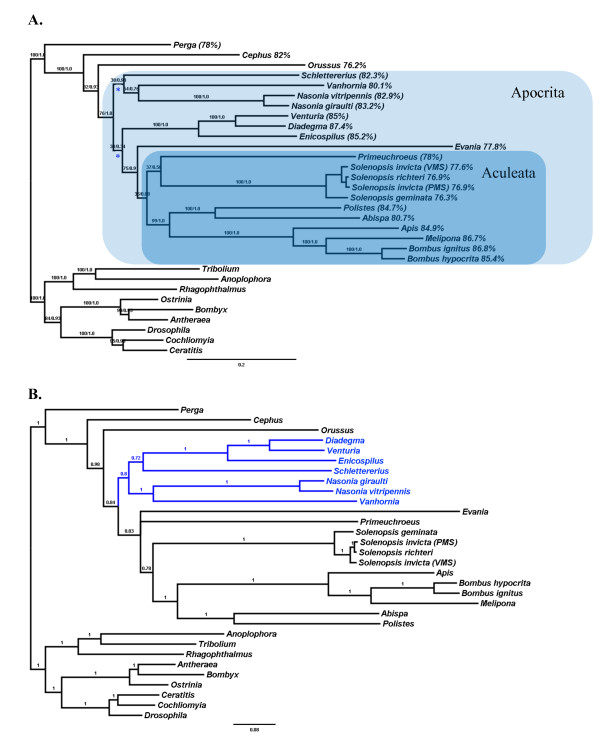
**Phylogenetic hypotheses of Hymenoptera based on protein coding genes and rRNA genes**. A. Phylogeny recovered from maximum likelihood analyses. Topology of the homogeneous (PhyML) and non-homogeneous (nhPhyML) analyses were identical. Values above internal branches are bootstrap (100 replicates) and aLRT (SH-like) branch support estimates. Values for percent A+T-bias are shown after genus name (values in parentheses are calculated from incomplete genome sequences). The apocritan and aculeatan clades are highlighted. The blue stars indicate posterior probabilities of 0.85 in the heterotachous Bayesian analysis (see B below); the other branches in this part of the tree were recovered with posterior probabilities of 1.0. B. Phylogeny derived from Bayesian inference. The tree topology between the homogenous and heterotachous analyses are identical, except for the clade highlighted in blue: the heterotachous analysis recovered phylogenetic relationships of these taxa which are identical to the maximum likelihood analyses shown in A. Bayesian posterior probabilities are given for the homogeneous model of nucleotide substitution, which is identical to that of the heterotachous model except for the clade in blue (see A above).

**Figure 5:**
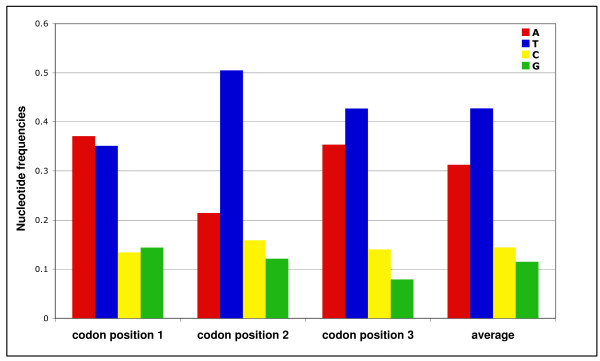
**Average nucleotide bias in protein coding genes averaged across all 4 *Solenopsis *mitogenomes**. There are no significant differences between mitogenomes (data not shown).

The four *Solenopsis *mitogenomes are consistently A+T-biased, but this bias is less pronounced than in any of the other apocritan Hymenoptera (~1-10% less; Figure 4A). Wei et al. [44] describe the same pattern for a distantly related evanid wasp, which suggests that the A+T-bias is perhaps more variable across the hymentoperan phylogeny than previously realized. There was no significant difference in nucleotide bias of the four ant mitogenomes (Table 1). Additionally, a T-bias persisted across protein coding regions on the coding strand (Figure 5). This was especially pronounced across the second codon positions. No evidence for codon bias was found after correcting for nucleotide bias (Table 1).

### Recombination

**Figure 6:**
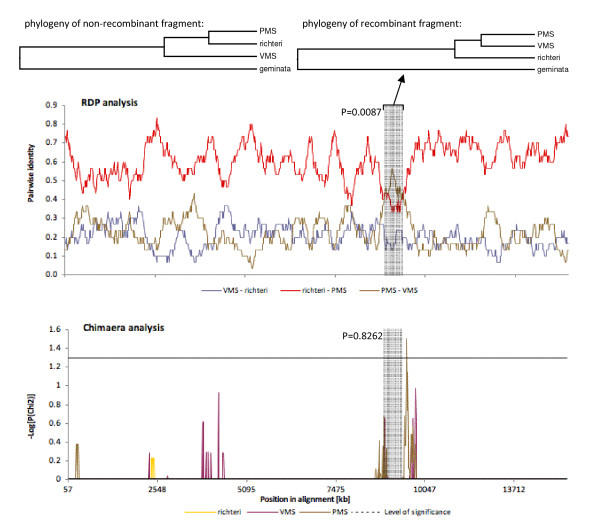
**Recombination graph of RPD (significant) and Chimaera (non-significant) analyses**. Regardless of the significance level, both show evidence of the same ~500 bp recombination event (grey shading) from the minor parent (VMS) to the recombinant daughter (PMS). The maximum likelihood phylogenies for the non-recombinant (left) and the recombinant (right) fragments are shown.

Recombination in animal mitogenomes has been well documented [45-47]. Nonetheless, such recombination remains difficult to detect reliably, no doubt in part due to the lack of power of many analytical methods developed for detecting recombination [48]. This pattern is reflected in our data set as well, as most methods were unable to detect statistically significant signatures of recombination. However, three local methods found significant support for a single recombination event within the PMS mitogenome (Table 3, Figure 6), with an approximately 500 bp surrounding the *nd6 *- *cytb *junction (nucleotide positions 8,971-9,517 of the aligned *Solenopsis *mitogenomes) derived from a VMS-like ancestor.

**Table 3:** Results of tests of recombination for the translocation of 500 bp fragment at nt8,950-9,483.

Program	Test	Probability	Type of test	**Ref**.
RDP3	RDP	**0.0087**	local	[103]
	GENECONV	*0.6320*	local	[104]
	BootScan	**0.0212**	local	[105]
	MaxChi	*0.3103*	local	[106]
	Chimaera	*0.8262*	local	[107]
	Sister Scan	*0.6969*	local	[108]
	3Seq	0.3514	--	[109]
	Distance plot	*yes*	local	
	PhylPro	*yes*	--	[110]
	LARD *	--	--	[111]
TOPALi2.5	DSS	no	local	[112]
	LRT	**YES**	local	
	PDM	no	--	[113]
	HMM	no	--	[114]
RecombiTest	GENECONV*	0.0929	global	[115]
	MaxChi *^,1^	0.85	global	[106]

Significant probability values are indicated in bold, statistically insignificant values that still show a pattern of recombination at that position are italicized (only applies to local methods). Asterices (*) mark methods used to verify recombination detected by other methods. ^1 ^was run on 5.5 kb window around the 500 bp recombinant.

Consistent with this scenario, six additional methods of analysis showed statistically non-significant yet congruent signals of recombination in the same location. One likely reason for the comparatively weak support for the hypothesized recombination event is that the translocation could have occurred sufficiently long ago that the signal of the event has decayed. Our results are unlikely an experimental artifact, since the recombinant sequence was recovered from separate PCR (and sequencing) reactions in which the initial PCR reactions utilized different conserved primers flanking this region. Further, the sequence data were high quality, the sequences differ slightly from the other three sequences, and the sequence traces do not show any evidence of conflicting base calls.

### Phylogeny

The phylogenetic tree recovered from all four *Solenopsis *mitogenomes is identical to a tree generated previously using sequence data from a much shorter region (coxI/tRNA-L_2_/coxII) and demonstrates that the *S. invicta *mitogenomes are not monophyletic: the PMS *invicta *mitogenome is more closely related to *richteri *than the VMS *invicta *mitogenome [49] (Figure 4). This phylogenetic pattern is upheld throughout the complete mitochondrial alignment, except for the short fragment shown above to have been transferred during the recombination event (Figure 6).

Our phylogenetic analysis of all hymenopteran mitogenomes encountered similar problems described previously [6,50-52] in that some expected relationships were not recovered, e.g. the sister taxa relationship between the Proctotrupomorpha (*Nasonia *and *Vanhornia*; *sensu *Rasnitsyn [53]) and the remainder of the Apocrita [50,54] or the sister relationship of the Ichneumonoidea (represented by Ichneumonidae in this study) with the Aculeata (Vespomorpha *sensu *Rasnitsyn [53]) [6,51,52,54]. Also, our phylogenetic analyses were unable to support the monophyly of the Vespoidea (ants and vespids; Figure 4) due to the placement of *Solenopsis *outside of the Vespidae + Apoidea clade with high branch support. While ants generally are considered to belong to the Vespoidea [55], some doubts have been raised regarding the monophyly of this superfamily [54,56] and our results mirror those of Dowton et al. [52] using only the mitochondrial 16 S rRNA locus.

Our homotachous (site specific rates do not change along tree) Bayesian analysis recovered a topology fully concordant with that of Dowton et al. [6] (Figure 4B). This was not surprising, since we tried to follow the suggestions made by Dowton et al. [6] regarding the best analysis parameters for hymentoperan mitogenomic data (i.e., using nucleotide data, exclusion of 3^rd ^codon positions, sampling several outgroups, and using a partitioned Bayesian framework). This suggests that the phylogenetic inference was robust to additional taxon sampling (*Solenopsis*, *Diadegma*, *Evania*). However, this topology differed from our other analyses in the placement of Ichneumonidae. Whereas the homotachous Bayesian analysis recovered the Ichneumonidae as a sister group to *Schlettererius *(Stephanidae), the other analyses placed the Ichneumonidae basal to an Aculeata + *Evania *clade. The analyses also differed in the placement of *Primeuchroeus*. Both maximum likelihood methods placed *Primeuchroeus *with *Solenopsis*, whereas both Bayesian analyses left its position unresolved at the base of the other Aculeata. However, all of these placements were poorly supported regardless of the branch support measure. Our analyses suggest that optimality criteria and models of nucleotide evolution have a stronger influence on the resulting phylogenetic inference in our dataset than taxon sampling. The importance of optimality criterion was previously also noted by Dowton et al. [6] and suggests that the extraction of phylogenetic signal from hymenopteran mitogenomic data is not simple.

While Dowton et al. [6] showed that the accuracy of mitogenomic phylogenetic analysis is greatly improved by the implementation of a particular partitioning scheme in a Bayesian framework, their analyses failed to account for heterotachous (site specific rate of evolution changes along the tree) and non-stationarity (base frequencies change along the tree) substitution processes between branches during phylogenetic analyses [57-60], of which the latter is an especially well-known feature of the hymenopteran mitogenomes [[6,44,61,62] this study]. Hence, we compared the phylogenies derived under homotachous and stationary models of nucleotide substitution with those allowing site specific rates of change and base frequencies to change between branches. We implemented the covarion model [59] in MrBayes to accommodate heterotachy and the model of Galtier and Gouy [57] to account for non-stationarity (and non-homogeneity) using nhPhyML. Applying Galtier and Gouy's [57] model under the maximum likelihood criterion significantly improved the fit of the model to the data (nhPhyML: -lnL = 159,198.79453; PhyML: -lnL = 160,821.701853; df = 60, LRT = 3245.8125, p < 0.0001 [58]), but it did not result in a different topology (Figure 4A). Use of the covarion model (which accommodates heterotachy by allowing sites to change their substitution rate along branches by being switched on or off) in the Bayesian framework did not result in an improvement in log-likelihood (covarion model: -lnL = 156,475.84; stationary model: -lnL = 156,228.88), but recovered a different topology almost identical to that recovered under the maximum likelihood optimality criterion (Figure 4B). This decrease in log-likelihood of the covarion model was surprising [but see [60]], but the exact implementation of heterotachy may be important [60,63] so the evaluation of other heterotachous models (e.g., mixed branch length model [64] or the RERV model [65]) may be warranted. More thorough studies are undoubtedly required to tease apart the contribution and possible interaction between different models and optimality criteria [4,6,60,63,66].

It is evident that reconstructing the hymenopteran phylogeny using only mitogenome data is problematic due to a variety of factors, e.g, differences in GC-content and rates of evolution among branches [64] or an ancient rapid diversification event [67,68] which is known to greatly complicate phylogenetic inference [69]. To account for these confounding factors, one should attempt to break up long branches by increased taxon sampling wherever possible. Including several species per genus (*Nasonia*, *Bombus*, and *Solenopsis*) as we have done is not very effective. Hence we suggest adding more genera and previously unsampled higher level taxa to more effectively break up long branches. While increased taxon sampling will likely rapidly occur in the near future, we agree with earlier suggestions of the importance of not relying on mitogenomes as the sole source of data for inferring phylogenetic relationships [70]. Mitochondria essentially provide one very specific evolutionary history of a lineage since they are maternally inherited as single markers independently from the nuclear genome, and their utility can be further compromised if their transmission is influenced by intracellular symbionts such as *Wolbachia *[70]. Given the rapidly decreasing costs in high-through-put sequencing and the various rapidly increasing genomic resources for several apocritan lineages (*Apis *and *Nasonia *genomes [71-73], *Solenopsis*, *Camponotus*, *Vespula*, and *Microctonus *genomic libraries [74-78]), we are hopeful that future phylogenetic work will be based on a broader genetic basis employing many more molecular characters [72].

## Conclusions

To fully clarify the evolution of mitochondrial genomes in the Hymenoptera will require much greater sampling at all taxonomic levels (i.e., from the superfamily to the species and possibly intraspecific level), which should provide much needed data to fully address the patterns and rate of evolution and genome organization in this organelle. While this information will yield an increased understanding of hymenopteran evolutionary history, mitogenome studies need to be complemented by phylogenetic studies utilizing other sources of data against which we can compare and contrast the information yielded by whole mitochondrial genome analyses. Given the recent rate of publications with full mitogenomes, this will no doubt occur in the near future, yet could be greatly optimized through concerted sampling efforts by the greater scientific community.

## Methods

### Generation of sequence data

Complete mitochondrial genomes were generated for four individuals from three closely related *Solenopsis *species. The two *S. invicta *samples were collected in Pascagoula, MS (PMS) and Vera, MS (VMS), *S. geminata *was collected in Tallahassee, FL, and *S. richteri *was collected in Rosario, Argentina. Even though the two *S. invicta *mitogenomes were sequenced from specimens collected in the invasive range, the ~1 kb coxI/tRNA-L_2_/coxII section of these mitogenomes are identical to haplotypes from the native range (PMS is H22 [GenBank: AY249114]; VMS is H5 [GenBank: AY249097]) and represent two widely divergent clades within *S. invicta *and two geographically and genetically distinct populations (Argentina and Brazil) [49,79]. We sequenced the entire mitogenome of each individual using a primer walking approach by performing 34-40 separate PCRs of genomic portions of the genome of variable size (~400-1,000 bp). We designed primers such that substantial overlap occurs between the various amplicons (allowing independent verification of sequence data by increasing depth of coverage) and such that the combination of all PCR amplicons spanned the entire genome. We initially attempted to amplify portions of the genome using conserved primers published in Simon et al. [80] as well as by designing conserved primers for a subset of coding genes by aligning available mitogenome sequences for *Apis mellifera *[GenBank: NC001566] and *Drosophila yakuba *[GenBank: NC001322]. We subsequently designed additional primers spanning other regions with no or low coverage using sequence data generated for fire ants. All primers developed and used for this study are presented in Additional file 1.

Several lines of evidence suggest that our sequences generated specifically represent mitochondrial genomic DNA rather than nuclear mitochondrial-like sequences (numts), which appear to be common and are generally short and highly fragmented in ants and other Hymenoptera [81-83]: The redundant yet independent PCR amplification of essentially every genomic region, the absence of ambiguous base calls characteristic of heterozygotes, the PCR amplification of the complete genome, and the generation of a contig identical in sequence to PMS using 454 pyrosequencing technology (DDS unpublished data).

All PCR amplicons were sequenced in both directions and each strand was assembled into single contigs with overlapping ends, indicating that our mitogenome sequences contained no gaps. Leading and lagging strand for each mitogenome were then aligned and manually checked for indels or ambiguous base calls. Mitogenomes were deposited in the NCBI GenBank database [GenBank: HQ215537, HQ215538, HQ215539, HQ215540].

### Genome annotation

Mitogenomes were annotated using the DOGMA webserver [84], which uses BLASTX against a custom database to identify protein coding genes. We verified all annotations made with DOGMA: coding regions were checked against a *S. invicta *EST database [75] and tRNAs were validated using ARWEN 1.2 [85] and tRNAscan-SE 1.21 [86] since DOGMA only uses COVE [87] to identify tRNAs. Generally, tRNAscan-SE has very low false positive rates and thus rarely mispredicts tRNAs (COVE scores ≥ 20 are usually considered reliable [86]), whereas ARWEN has a low false negative rate and usually identifies all tRNAs [85]. Generally, DOGMA identified significantly more tRNAs than either ARWEN or tRNAscan-SE, sometimes with quite high COVE scores. Two tRNAs in particular were not recovered, tRNA-S_1 _and tRNA-N. These, however could be folded manually.

### Sequence analyses

Nucleotide sequences were aligned based on amino acid alignments using MUSCLE 3.6 [88]. Models of nucleotide evolution were estimated for protein coding genes using jModeltest [89]. DnaSP 4.50.3 [90] was used to estimate codon usage bias and nucleotide frequency bias [91-93]. The CODEML program in the PAML4.2 package [94] was used to test for site-specific evidence of positive selection while correcting for nucleotide bias [95]. We employed the following parameters: runmode = 0, omega and kappa estimated (from three different starting points), empirical codon frequencies from each codon position (codonfreq = 2).

Following the recommendations of Posada [48], we employed a suite of recombination detection programs offered in the program packages TOPALi 2.5 [96] and RPD 3b32 [97] and the RecombiTest website [46]http://www.lifesci.sussex.ac.uk/CSE/test/index.php to test for recombination in the *Solenopsis *mitogenomes (see Table 3 for specific tests used). When any of the recombination tests only utilized three sequences at a time (e.g., RDP), analyses were repeated with every possible sequence triplet combination and p-values were Bonferroni corrected. All settings were left at the software default for the initial analyses, except for the PDM and LRT, where we used flexible window sizes. The highest acceptable *p*-value was 0.05 (unless Bonferroni corrected). Loosely following Tsaousis et al.'s [45] criteria for evidence of recombination, we consider as good evidence for recombination when more than one test detected a recombination event (although without regard to the test being a global or a local method). The more tests recovering evidence for recombination the more confident we are that it represents a true recombination event. Although this classification is admittedly arbitrary, we agree with White et al. [98] that identifying instances of recombination is inherently difficult and requires the heuristic use of several methods to identify potential recombinants.

Phylogenetic analyses were conducted on protein coding genes of the hymentoperan mitogenomes and 9 outgroups (3 flies [GenBank: X03240, AF260826, AJ242872], 3 beetles [GenBank: AJ312413, DQ768215, AB267275], and 3 moths [GenBank: AF442957, AF149768, AY242996]). jModeltest [89] was used to estimate the most appropriate model of nucleotide evolution for each codon position at each locus separately. Following the suggestion of Dowton et al. [6] we used the Bayesian approach using nucleotide sequences and implemented the GTR+I+Γ model of sequence evolution across genes and codon positions since jModeltest usually indentified this model as the best fitting for each data partition. MrBayes 3.1.2 [99] was then used to recover phylogenetic hypotheses. All parameters were unlinked between partitions. Two independent analyses were run for three million generations, each with three heated and one cold chain. Parameters were sampled every 1000^th ^generation. Convergence between runs was assessed when log-likelihoods had plateaued, PRSF factors were ~1, and split frequencies had dropped < 0.01. Samples taken prior to convergence were removed before samples were summarized. The same analysis was repeated implementing the covariotide model of sequence evolution to account for heterotachy (changes in site-specific evolutionary rates across lineages) [59], which has been shown to effectively accommodate heterotachy [[66], but see [60]]. Since this analysis took longer to converge, 5 million generations were run.

Maximum likelihood analyses were implemented on the PhyML 3.0 webserver [100]http://www.atgc-montpellier.fr/phyml/. We implemented the GTR+I+Γ model of nucleotide substitution on the unpartitioned dataset, estimated proportion of invariable sites and gamma shape parameter using six substitution rate categories, and optimized equilibrium frequencies, branch lengths, and tree topology (using the nearest-neighbor interchange [NNI] and sub-tree pruning and regrafting [SPR]) on five random starting trees. In addition to running one hundred bootstrap replicates to estimate levels of branch support, we also implemented the SH-like aLRT, which assesses the likelihood gain of the presence of that branch [101]. To accommodate non-stationarity (changes of base frequencies between branches) we implemented nhPhyML-Discrete [58] using default options and the topology recovered from the heterotachous Bayesian analysis as the starting tree.

The evolution of tRNA-N was studied using phylogenetic analyses as suggested by Saks et al. [34] and Dowton and Austin [29], which were conducted using 100 bootstrap replicates in PhyML using the same configuration as described above. Other relevant hymenopteran tRNAs (D, N, and V) were downloaded from GenBank and aligned using MUSCLE. Unlike other authors [29,37,38], unpaired loops and anticodons were not removed following the suggestions of Wong et al. [102]. However, we would like to point out that the phylogenetic analysis should only be interpreted as a heuristic tool, since the alignment of many very short, evolutionary very old, and highly AT-biased sequences is not trivial, regardless of alignment method used or prior editing to remove problematic areas.

## Authors' contributions

JC and DDS carried out all aspects of the molecular lab work. DG and DDS performed all analyses of the data and wrote the manuscript. All authors read and approved the final manuscript.

## Supplementary Material

Additional file 1 **Primers used for PCR amplification and sequencing of fire ant genomes**. Primers names and sequences used in this study. J and N within primer names refer to heavy and light strands, respectively, and indicate the orientation of the primers. Primer names beginning with "Gem" were designed specifically to amplify the mitogenome of *S. geminata*. Click here for file
